# Sequential Screening of Lactic Acid Bacteria from Fermented Sour Bamboo Shoots and Their Effects on Patient-Derived Fecal Microbiota In Vitro

**DOI:** 10.3390/microorganisms14092019

**Published:** 2026-09-11

**Authors:** Junjie Li, Lianxian Ma, Rui Shi, Yuanrui Yu, Wenyan Zhang, Peng Zhu, Kaijie Kang, Liwen Jiang

**Affiliations:** 1 School of Chemistry and Chemical Engineering, Zhaotong University, Zhaotong 657000, China; lijunjie900501@163.com (J.L.);; 2 College of Food Science and Technology, Hunan Agricultural University, Changsha 410128, China

**Keywords:** gastrointestinal tolerance, antibiotic susceptibility, short-chain fatty acids, gut microbiota, pathogen antagonism

## Abstract

Naturally fermented sour bamboo shoots represent a potential source of functionally active lactic acid bacteria (LAB). This study screened LAB from sour bamboo shoots and evaluated their potential to modulate patient-derived fecal microbiota in vitro. A total of 42 candidate isolates were obtained by morphological and physiological/biochemical screening. Five isolates showing intermediate antibiotic resistance were excluded, and the remaining 37 isolates were evaluated for gastrointestinal tolerance, antagonistic activity against enteric pathogens, and adhesion-related properties. Principal component analysis (PCA) identified nine strains for further evaluation. These strains were individually introduced into an in vitro colonic fermentation system prepared from pooled fecal samples of patients with bacterial diarrheal enteritis. After 48 h of fermentation, microbial community composition was assessed by 16S rRNA gene sequencing, and short-chain fatty acids (SCFAs) were quantified. LAB treatment was associated with a higher relative abundance of Firmicutes and a lower relative abundance of Proteobacteria. Compared with the mixed fecal suspension (MFS) control, the relative abundance of *Escherichia–Shigella* decreased to below 1% in the MRS-6, MRS-12, MRS-36, and MRS-46 groups, accompanied by increases in health-associated commensal genera including Blautia, Bifidobacterium, Faecalibacterium, and Bacteroides. Total SCFA concentrations reached 14.51, 13.55, and 12.16 mmol/L in the MRS-36, MRS-46, and MRS-6 groups, respectively, with the highest butyrate concentrations observed in the MRS-36 and MRS-46 groups. These findings establish a sequential screening framework linking in vitro functional phenotypes with validation in a complex fecal microbial community and identify candidate strains with promising microbiota- and SCFA-modulating potential.

## 1. Introduction

Naturally fermented foods are important sources of lactic acid bacteria (LAB) and other microorganisms with potential probiotic properties. Whether an isolate can be further developed as a probiotic depends not only on its taxonomic identity but also on its survival under gastrointestinal conditions, safety profile, adhesion-related phenotypes, and antagonistic activity against pathogenic microorganisms [1,2,3]. Previous studies have emphasized that probiotic evaluation should encompass survival to the target site, interactions with the host or resident microbiota, antipathogenic properties, and safety [4].

Sour bamboo shoots are a traditional salt-free fermented food produced by the natural fermentation of fresh bamboo shoots in water. Lactic acid bacteria become progressively enriched during fermentation, making this food a potential reservoir of functionally relevant LAB [5]. However, studies on LAB derived from sour bamboo shoots have largely focused on isolation, identification, or individual in vitro traits, whereas their safety, integrated physiological properties, and behavior within complex gut microbial communities remain insufficiently characterized [6].

Safety assessment should precede functional screening of LAB. Strains showing potential risks, such as atypical antibiotic resistance, hemolytic activity, or mucin degradation, should first be excluded, followed by evaluation of tolerance to gastric acid, bile salts, and digestive conditions, as well as pathogen antagonism, autoaggregation, coaggregation, and cell-surface hydrophobicity [7,8]. Because individual strains often perform differently across these traits, selection based on a single parameter may not adequately reflect their overall functional potential. Multivariate approaches such as principal component analysis (PCA) can therefore provide a more integrated assessment. Nevertheless, conventional antimicrobial and tolerance assays mainly reflect strain behavior under simplified experimental conditions and cannot fully reproduce the competition, cooperation, and metabolic interactions that occur within the intestinal microbiota [9]. In vitro fecal fermentation systems that preserve complex microbial communities, combined with high-throughput 16S rRNA gene sequencing, provide a useful approach for further evaluating candidate strains by examining microbial diversity, community structure, and changes in the relative abundance of target taxa [10,11].

Accordingly, LAB were isolated from naturally fermented sour bamboo shoots and subjected to physiological/biochemical characterization and 16S rRNA gene-based identification, followed by sequential assessment of safety, pathogen antagonism, simulated gastrointestinal tolerance, and adhesion-related phenotypes. PCA was then used to identify strains with favorable overall performance. A pooled fecal inoculum from patients with bacterial diarrheal enteritis was subsequently used to establish an in vitro fermentation system to evaluate the effects of the selected strains on the relative abundance of Escherichia–Shigella and on overall microbial community composition.

## 2. Materials and Methods

### 2.1. Sour Bamboo Shoot Samples and Strain Isolation

Bamboo shoots were collected in Liuzhou, Guangxi, China (24°45′12″ N, 109°25′12″ E) and transported to the laboratory within 24 h of harvest. After removal of the outer sheath and washing with clean water, the edible middle portion was cut into strips approximately 5 cm long and 0.5 cm in both width and thickness and placed in a sterile 15 L glass jar. Distilled water that had been boiled and cooled to room temperature was added until the liquid level was approximately 5 cm above the samples. The jar was sealed and incubated for natural anaerobic fermentation at 20–25 °C for 15 d. A 25 g portion of fermented sour bamboo shoots was homogenized in 225 mL of sterile 0.85% saline and serially diluted from 10^−2^ to 10^−7^. Aliquots (200 μL) from each dilution were spread onto MRS and M17 media supplemented with 2% CaCO_3_ and onto MC medium, followed by incubation at 37 °C for 48 h. Single colonies surrounded by clear zones were selected, and Gram-positive isolates were retained after Gram staining. Candidate isolates were purified by three successive rounds of streaking, transferred to the corresponding agar slants, and stored at 4 °C until use. Cell morphology was observed under oil immersion after Gram staining. Preliminary identification was performed according to Bergey’s Manual of Determinative Bacteriology [12].

### 2.2. 16S rRNA Gene Amplification, Sequencing, and Strain Identification

Genomic DNA was extracted using a TSINGKE DNA extraction kit (Tsingke Biotechnology Co., Ltd., Beijing, China). The bacterial 16S rRNA gene was amplified using the universal primers 27F (5′-AGAGTTTGATCMTGGCTCAG-3′) and 1492R (5′-GGTTACCTTGTTACGACTT-3′). The polymerase chain reaction (PCR) program consisted of an initial denaturation at 98 °C for 2 min, followed by 35 cycles of 98 °C for 10 s, 55 °C for 10 s, and 72 °C for 10 s·kb^−1^, with a final extension at 72 °C for 5 min. PCR products were verified by agarose gel electrophoresis and subjected to bidirectional Sanger sequencing. The resulting sequences were assembled in ContigExpress (version 11.5.0), low-quality regions were removed, and the curated sequences were compared against the NCBI (Bethesda, MD, USA) database using BLAST (https://blast.ncbi.nlm.nih.gov/Blast.cgi, accessed on 7 September 2026) for preliminary taxonomic assignment.

### 2.3. Preliminary Phenotypic Safety Screening

(1) Preliminary phenotypic antibiotic susceptibility screening

Antibiotic susceptibility was preliminarily evaluated using the disk diffusion method with 0.6-cm filter-paper disks. The antibiotics tested included ampicillin (10 μg/disk), amoxicillin (10 μg/disk), levofloxacin (10 μg/disk), chloramphenicol (30 μg/disk), erythromycin (15 μg/disk), clindamycin (20 μg/disk), tetracycline (30 μg/disk), vancomycin (30 μg/disk), cefoperazone (20 μg/disk), gentamicin (10 μg/disk), tobramycin (10 μg/disk), and streptomycin (300 μg/disk). Inhibition-zone diameters were measured in millimeters after incubation. Because species- and antimicrobial-specific Clinical and Laboratory Standards Institute (CLSI) disk-diffusion breakpoints are not uniformly available for all LAB taxa and antibiotics examined in this study, the inhibition-zone thresholds used here were not interpreted as CLSI clinical breakpoints. Instead, based on previously reported disk-diffusion screening approaches for LAB [13,14] inhibition-zone diameters were operationally categorized as >2.0 cm, 1.5–2.0 cm, and ≤1.4 cm for preliminary phenotypic screening. These categories were used only to identify isolates showing comparatively reduced susceptibility and to prioritize strains with a more favorable preliminary safety profile for subsequent functional evaluation. Definitive antimicrobial susceptibility assessment would require species- and antimicrobial-specific minimum inhibitory concentration (MIC) determination and, where appropriate, genomic analysis of antimicrobial-resistance determinants.

(2) Mucin Degradation Activity

Glucose-free MRS, M17, and MC media containing 1.2 g/100 mL agar were supplemented with 0.5 g/100 mL type III porcine gastric mucin. Activated bacterial suspensions (100 μL) were spread onto the corresponding media and incubated at 37 °C for 48 h. The plates were then stained with 0.1% (*w*/*v*) amido black, and 1.2 mol/L acetic acid was applied to the bacterial lawn. The appearance of a white, cloud-like precipitate against the dark background was recorded as a positive reaction; absence of such a precipitate was considered negative.

(3) Hemolytic Activity

Purified isolates were streaked onto Columbia blood agar plates and incubated at 37 °C for 18–24 h. Hemolysis was assessed according to colony-associated zones: α-hemolysis was defined by a narrow greenish zone, β-hemolysis by a broad, sharply demarcated transparent zone, and γ-hemolysis by the absence of a hemolytic zone.

### 2.4. Evaluation of In Vitro Physiological and Functional Properties

(1) Determination of Antagonistic Activity

Antagonistic activity was evaluated using a modified Oxford cup diffusion assay [15] against *Escherichia coli* (CMCC(B) 43201), Shigella dysenteriae (BNCC 103609), and Vibrio parahaemolyticus (CMCC(B) 20033). A sterile 2% agar solution was poured as the basal layer of each Petri dish. After solidification, sterile Oxford cups (8 mm in diameter) were placed on the agar. Indicator bacterial suspensions at approximately 1 × 10^8^ colony-forming units (CFU)/mLwere mixed with nutrient agar and poured into the dishes. After solidification, the cups were removed and 200 μL of candidate-strain fermentation broth (1 × 10^8^ CFU/mL) was added to each well. Plates were incubated at 37 °C for 36 h, and the width of the clear inhibition zone extending outward from the edge of the cup was measured with a vernier caliper. An inhibition-zone width > 0.1 cm was considered indicative of antagonistic activity.

(2) Determination of Tolerance to Simulated Gastric and Intestinal Conditions

The assay was performed with modifications to a previously described method [16]. Candidate strains were activated in their corresponding MRS, M17, or MC media, washed twice with phosphate-buffered saline (PBS) (pH 7.2), and resuspended to approximately 1 × 10^9^ CFU/mL. The bacterial suspension was inoculated at 10% (*v*/*v*) into 100 mL (Final volume) of simulated gastric juice containing 0.85% NaCl and 3.0 mg/mL pepsin, adjusted to pH 2.5. After thorough mixing, cultures were incubated at 37 °C in a water-bath shaker under low-speed continuous agitation for 4 h, and viable counts were determined by plate counting.

Twenty milliliters of the gastric juice–cell mixture obtained after the gastric phase was adjusted to pH 7.2 with sterile 0.5 mol/L sodium bicarbonate buffer. Five milliliters of 0.6% trypsin solution passed through a 0.45 μm sterile filter and 5 mL of 1.8% (*w*/*v*) bovine bile salt solution were then added. The pH was readjusted to 7.2, and the mixture was incubated at 37 °C in a water-bath shaker under low-speed continuous agitation. Strain survival was determined after 24 h.

Survival rate (%) = (N_1_/N_0_) × 100%,

where N_0_ is the viable cell count at 0 h of simulated gastric or intestinal treatment, and N_1_ is the viable cell count after the indicated treatment period.

(3) Autoaggregation

Candidate strains activated for 16 h were harvested by centrifugation (10,000 rpm, 10 min, 4 °C), washed three times with sterile PBS (pH 7.0), and resuspended in sterile PBS to approximately 1 × 10^9^ CFU/mL. The suspensions were incubated at 37 °C for 1 h. Without disturbing the suspension, the absorbance of the upper phase at 600 nm was measured after 1 h using an ultraviolet–visible spectrophotometer [17,18].

Autoaggregation (%) = [(A_0_ − A)/A_0_] × 100%

where A_0_ is the initial absorbance and A is the absorbance after 1 h of incubation.

(4) Coaggregation

Coaggregation was measured using a modified method based on reference [17]. Cell suspensions of each candidate strain and *E. coli* were prepared as described for the autoaggregation assay. The optical density at 600 nm (OD_600_) values of the candidate-strain and *E. coli* suspensions were adjusted to 0.25 and 0.60, respectively. Equal volumes (5 mL each) of the two suspensions were mixed by vortexing for 10 s. After incubation at 37 °C for 5 h, the absorbance of the upper phase at 600 nm was measured. Coaggregation was calculated as follows:

Coaggregation (%) = [1 − 2A_mix_/(A_1_ + A_2_)] × 100%

where A_1_ and A_2_ are the absorbance values of the candidate strain and *E. coli*, respectively, at 0 h, and A_mix_ is the absorbance of the mixed suspension after 5 h of incubation.

(5) Cell-Surface Hydrophobicity

Activated cultures grown at 37 °C for 48 h were centrifuged at 10,000 rpm for 8 min at 4 °C and washed twice with sterile PBS (pH 7.0). Cells were resuspended in sterile PBS and adjusted to an OD_600_ of approximately 1.0. A 3 mL aliquot of the suspension was mixed with 0.6 mL xylene by vortexing for 2 min and then incubated at 37 °C for 30 min to allow phase separation. The aqueous phase was collected, and its OD_600_ was measured [19]. Cell-surface hydrophobicity was calculated as follows:

Cell-surface hydrophobicity (%) = [(A_0_ − A_1_)/A_0_] × 100%

where A_0_ is the OD_600_ of the initial cell suspension and A_1_ is the OD_600_ of the aqueous phase after extraction with xylene.

### 2.5. Comprehensive Screening Based on Principal Component Analysis

Principal component analysis (PCA) was performed using IBM SPSS Statistics. Eight variables were included: survival after 4 h in simulated gastric juice, survival after 24 h in simulated intestinal fluid, antagonistic activity against the three indicator pathogens, cell-surface hydrophobicity, autoaggregation, and coaggregation. PCA was conducted using the correlation matrix, and data suitability was evaluated using the Kaiser–Meyer–Olkin (KMO) test and Bartlett’s test of sphericity. Principal components with eigenvalues > 1 were retained. A comprehensive score was calculated according to the variance contribution of each retained component, and the strains were ranked in conjunction with the safety-screening results. Strains with favorable overall performance were selected for subsequent in vitro fecal fermentation.

### 2.6. In Vitro Fecal Fermentation Using Samples from Patients with Bacterial Diarrheal Enteritis

Fresh fecal samples were obtained from the Clinical Laboratory Department of Yueyang Hospital of Traditional Chinese Medicine. Five volunteers diagnosed with bacterial diarrheal enteritis were enrolled. All participants followed a habitual Chinese diet, had not received antibiotics for at least 1 month before sampling, provided informed consent, and were enrolled under a protocol approved by the Biomedical Research Ethics Committee of Hunan Agricultural University. The participants were a 45-year-old man, a 20-year-old man, a 66-year-old man, a 25-year-old woman, and a 54-year-old woman. Equal amounts of feces from the five participants were pooled to generate a composite fecal inoculum. The pooled feces were suspended in nine volumes of sterile PBS to prepare a 10% (*w*/*v*) mixed fecal suspension (MFS) and filtered through four layers of sterile gauze. The fecal slurry was mixed with sterile colonic culture medium to prepare a 10% fecal fermentation suspension (Simulated colonic medium (per 1 L) contained 8.0 g starch, 3.0 g tryptone, 3.0 g peptone, 4.5 g yeast extract, 4.5 g NaCl, 0.4 g K_2_HPO_4_, 0.4 g KH_2_PO_4_, 0.45 g MgSO_4_·7H_2_O, 0.2 g CaCl_2_·6H_2_O, 1.5 g NaHCO_3_, 0.05 g hemin, 0.02 g vitamin K_1_, 0.8 g L-cysteine hydrochloride, 0.4 g bile salts, 1 mL Tween 80, and 0.25 g resazurin. The pH was adjusted to 6.8 ± 0.2). Candidate-strain suspensions containing 1 × 10^8^ CFU were added individually at a 1% inoculation volume; for the MFS group, add an equal amount of sterile colon culture medium. Fermentation was performed anaerobically at 37 °C for 48 h under an N_2_:H_2_:CO_2_ atmosphere of 85:10:5. Microbial community composition and short-chain fatty acid (SCFA) concentrations were evaluated as complementary endpoints reflecting compositional and metabolic responses, respectively, to candidate-strain supplementation.

### 2.7. 16S rRNA Gene Amplicon Sequencing and Microbiota Analysis

Total microbial DNA was extracted from fecal samples using a magnetic bead-based fecal genomic DNA extraction kit (BioTeke, Beijing, China) and quantified using Qubit (Invitrogen, Carlsbad, CA, USA). The V3–V4 region of the bacterial 16S rRNA gene was amplified using primers 341F/805R. PCR products were purified using AMPure XT beads (Beckman Coulter Genomics, Danvers, MA, USA) and subjected to library quality control, followed by 2 × 250 bp paired-end sequencing on an Illumina NovaSeq 6000 platform. Raw reads were processed using Cutadapt (v1.9), FLASH (v1.9), fqtrim (v0.94), and VSEARCH (v2.3.4) for primer removal, read merging, quality filtering, and chimera removal. Divisive Amplicon Denoising Algorithm 2 (DADA2) was subsequently used for denoising and generation of an amplicon sequence variant (ASV) table. Alpha- and beta-diversity analyses were conducted in Quantitative Insights Into Microbial Ecology 2 (QIIME 2), and taxonomic annotation and relative-abundance profiling were performed using the SILVA and NT-16S databases. Sparse analysis was conducted separately for the four independent fermentation replication experiments within each treatment group. The average alpha diversity values at each subsampling depth were calculated for each replication experiment, and the resulting average sparse curves were plotted for each treatment group. Alpha-diversity indices, including Chao1, observed ASVs, Shannon, and Simpson indices, were calculated for each independent fermentation replicate after normalization to a consistent sequencing depth. Rarefaction curves were generated primarily to evaluate sequencing sufficiency and were not used for statistical inference among treatment groups. Differences in alpha-diversity indices among treatment groups were evaluated using the Kruskal–Wallis test, followed, where appropriate, by Dunn’s pairwise comparisons with Benjamini–Hochberg false discovery rate (FDR) correction.

### 2.8. Determination of Short-Chain Fatty Acids in Fecal Fermentation Samples

Fresh fecal fermentation broth was mixed with sterile PBS (1:9, *v*/*v*), centrifuged at 10,000 rpm for 8 min at 4 °C, and filtered through a 0.22 μm membrane. A 0.1 mL aliquot of the filtrate was brought to 1.0 mL with methanol, homogenized at low temperature, sonicated on ice for 10 min, and incubated at −20 °C for 30 min. After vortexing and centrifugation at 12,000 rpm for 5 min at 4 °C, the supernatant was appropriately diluted. A 100 μL aliquot was derivatized with pyridine, EDC, and 3-NPH and analyzed by LC–MS/MS. Separation was performed on an ACQUITY UPLC BEH C8 column (1.7 μm, 3.0 × 100 mm) at 40 °C, with a flow rate of 0.40 mL/min and an injection volume of 2 μL. Mobile phases A and B were 0.1% (*v*/*v*) formic acid in ultrapure water and acetonitrile, respectively. The gradient was: 0–2.0 min, 20% B; 2.0–8.0 min, 20–55% B; 8.0–8.2 min, 55–95% B; 8.2–9.2 min, 95% B; 9.2–9.5 min, 95–20% B; and 9.5–11.0 min, 20% B. Detection was performed in ESI−-MRM mode. Acetate, propionate, butyrate, isobutyrate, valerate, isovalerate, and hexanoate were quantified by external-standard calibration (0.1–200 μmol/L; R^2^ ≥ 0.995), and results were expressed as mmol/L. Solvent blanks, calibration standards, and pooled quality-control (QC) samples were included throughout the sequence. QC samples were analyzed before the batch, every ~10 samples, and at the end; a relative standard deviation (RSD) ≤ 15% was considered acceptable. Samples outside the calibration range or failing QC criteria were diluted or reprocessed and reanalyzed.

### 2.9. Statistical Analysis

All quantitative experiments were performed with four independent replicates (*n* = 4), and data are presented as mean ± standard deviation (SD). For fecal fermentation experiments, replicates were independently prepared from the same pooled fecal inoculum and were treated as independent fermentation replicates. Technical replicates were not considered independent observations. Normality and homogeneity of variance were assessed using the Shapiro–Wilk and Levene’s tests, respectively. For in vitro functional parameters and SCFA concentrations meeting these assumptions, differences among groups were analyzed by one-way analysis of variance (ANOVA) followed by Duncan’s multiple range test. Different lowercase letters indicate significant differences at *p* < 0.05. Alpha-diversity indices (Chao1, observed ASVs, Shannon, and Simpson) were compared using the Kruskal–Wallis test followed, where appropriate, by Dunn’s test with FDR correction. Good’s coverage was used descriptively to assess sequencing completeness. Relative taxonomic abundances were compared among treatment groups using the Kruskal–Wallis test based on replicate-level relative-abundance data. When a significant overall group effect was detected, Dunn’s pairwise comparisons were performed using the MFS group as the reference. Benjamini–Hochberg false discovery rate (FDR) correction was applied to account for multiple comparisons, and *q* < 0.05 was considered statistically significant. The hierarchical clustering heatmap was used for visualization of abundance patterns only, whereas statistical inference was based on the underlying replicate-level relative-abundance data. Beta diversity was assessed using Bray–Curtis dissimilarities. principal coordinates analysis (PCoA) was used for visualization, and permutational multivariate analysis of variance (PERMANOVA) with 999 permutations was used to test differences in community composition, with pseudo-F, R^2^, and *p*-values reported. PCA of the in vitro functional characteristics was performed using IBM SPSS Statistics version 26.0.0 after standardization of variables, *p* < 0.05 was considered statistically significant, and *q* < 0.05 was used for FDR-adjusted analyses.

## 3. Results

### 3.1. Isolation and Purification of Candidate Strains

A total of 148 isolates were recovered from fermented sour bamboo shoots through culture-based isolation and preliminary physiological/biochemical characterization: 59 isolates on MRS medium (MRS-1 to MRS-59), 48 on M17 medium (M17-1 to M17-48), and 41 on MC medium (MC-1 to MC-41). Based on preliminary identification according to Bergey’s Manual of Determinative Bacteriology [12], isolates showing a positive gelatin test, positive indole test, positive hydrogen sulfide test, negative methyl red test, or negative lactose-fermentation test were excluded. This yielded 42 candidate isolates: MRS-1, MRS-6, MRS-12, MRS-13, MRS-14, MRS-16, MRS-18, MRS-19, MRS-22, MRS-25, MRS-27, MRS-34, MRS-36, MRS-46, MRS-49, MRS-51, M17-2, M17-3, M17-4, M17-5, M17-6, M17-7, M17-8, M17-10, M17-14, M17-18, M17-22, M17-24, M17-29, M17-30, M17-36, M17-38, MC-1, MC-4, MC-5, MC-10, MC-14, MC-17, MC-19, MC-24, MC-30, and MC-38. The recovery of numerous isolates on all three media indicated that fermented sour bamboo shoots harbor diverse cultivable LAB. The marked reduction in strain number after physiological and biochemical screening also showed that culture-based isolation alone was insufficient for effective selection of candidate functional strains and that molecular identification and functional evaluation were required for stepwise screening.

### 3.2. Identification of Candidate Strains

**Figure 1:**
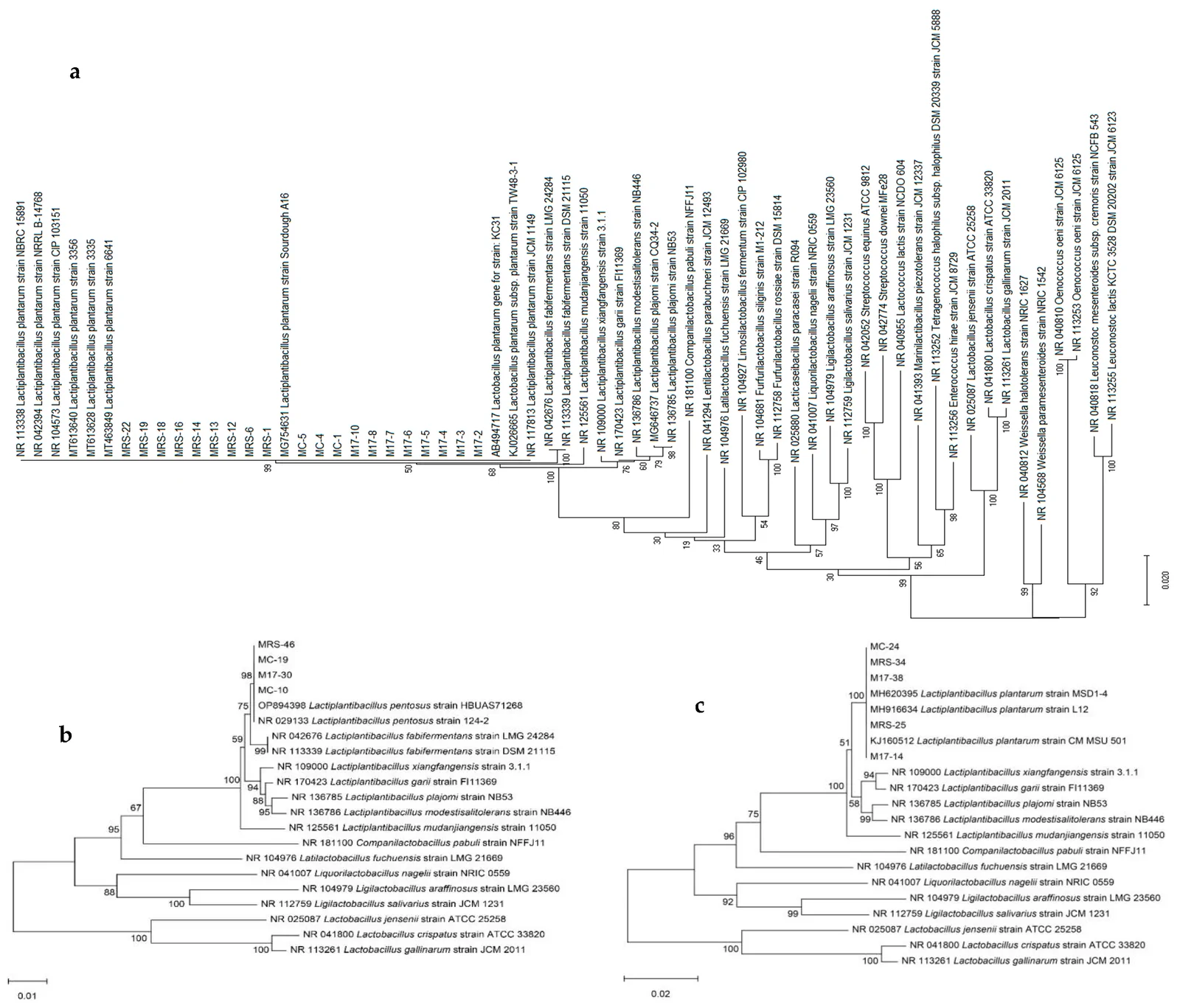

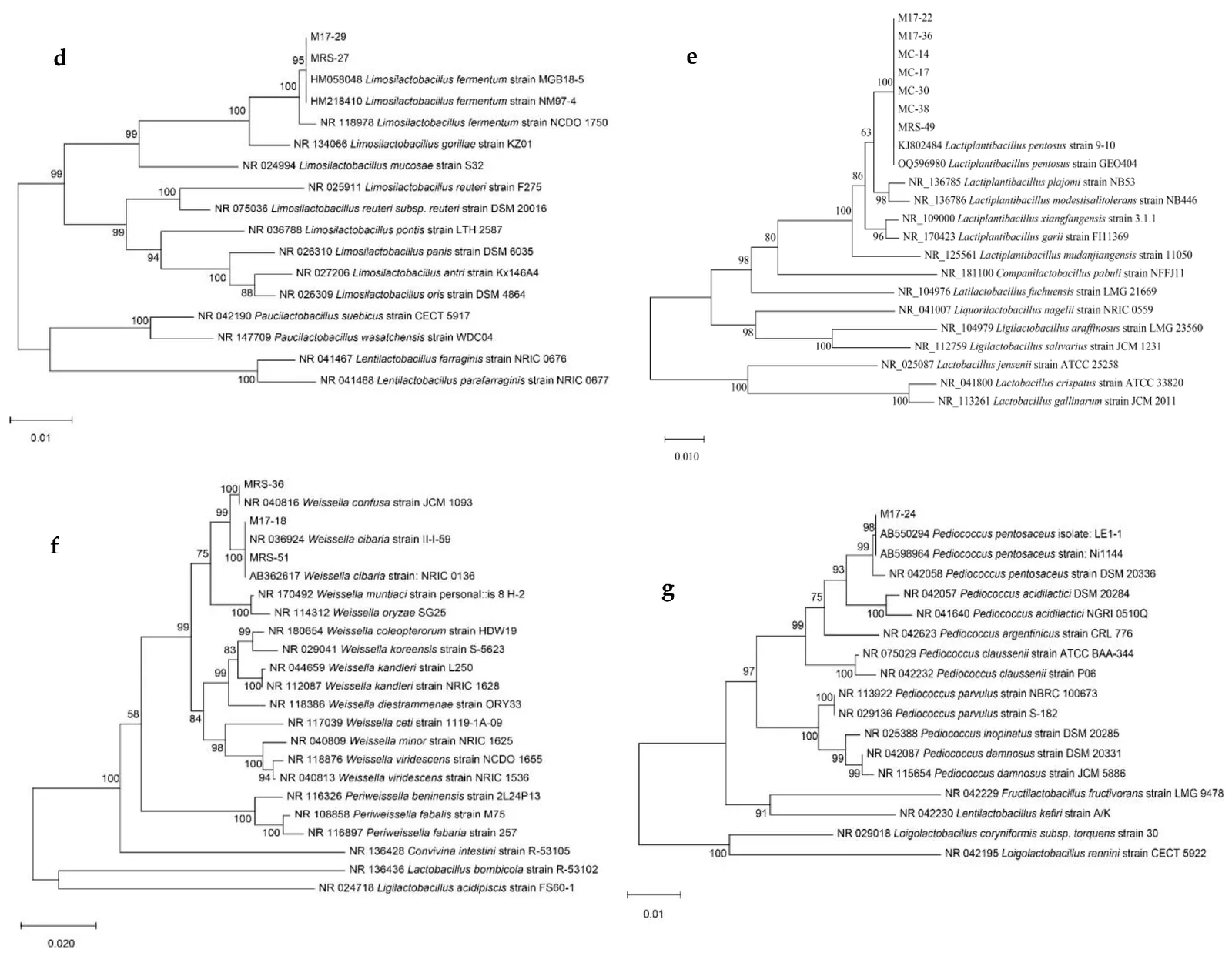
Phylogenetic trees of candidate lactic acid bacteria based on 16S rRNA gene sequences: (**a**,**b**) *Lactiplantibacillus plantarum* strains; (**c**,**d**) *Lactiplantibacillus pentosus* strains; (**e**) * Limosilactobacillus fermentum*; (**f**) *Weissella confusa*; and (**g**) *Pediococcus pentosaceus*. The numbers at the branch nodes represent bootstrap support values (%) based on 1000 replicates.

The 16S rRNA genes of the isolates were amplified and sequenced, and the resulting sequences were compared with reference sequences using BLAST. Phylogenetic trees were constructed using ClustalX 1.8 and MEGA 7. As shown in Figure 1a,b, MRS-1, MRS-6, MRS-12, MRS-13, MRS-14, MRS-16, MRS-18, MRS-19, MRS-22, M17-2, M17-3, M17-4, M17-5, M17-6, M17-7, M17-8, M17-10, MC-1, MC-4, and MC-5 showed 99% sequence similarity to *Lactiplantibacillus plantarum* strain Sourdough A16 (MG 754631), whereas MRS-25, MRS-34, M17-14, M17-38, and MC-24 showed 100% similarity to *L. plantarum* strain MSD1-4 (MH620395) and were assigned to *L. plantarum*. As shown in Figure 1c,d, MRS-49, M17-22, M17-36, MC-14, MC-17, MC-30, and MC-38 showed 100% similarity to *Lactiplantibacillus pentosus* strain 9-10 (KJ 802484), while MRS-46, M17-30, MC-10, and MC-19 showed 98% similarity to *L. pentosus* strain HBUAS 71268 (OP 894398); these isolates were assigned to *L. pentosus*. As shown in Figure 1e, MRS-27 and M17-29 showed 95% similarity to *Limosilactobacillus fermentum* strain MGB18-5 (HMO 58048) and were therefore tentatively assigned to *L. fermentum*. As shown in Figure 1f, MRS-51 and M17-18 showed 100% similarity to *Weissella cibaria* strain II-I-59 (NR 036924), and MRS-36 showed 100% similarity to *Weissella confusa* strain JCM 1093 (NR 040816); accordingly, MRS-51 and M17-18 were assigned to *W. cibaria* and MRS-36 to *W. confusa*. Finally, M17-24 showed 98% similarity to *Pediococcus pentosaceus* isolate LE1-1 (AB 550294) (Figure 1g) and was assigned to *P. pentosaceus*.

The present results show that naturally fermented sour bamboo shoots contained not only common fermentative LAB such as *L. plantarum* and *L. pentosus* but also members of several other LAB genera, indicating substantial taxonomic diversity. The recovery of strains from different taxa provided a basis for subsequent comparison of safety, gastrointestinal tolerance, pathogen antagonism, and microbiota-modulating effects.

### 3.3. Preliminary Phenotypic Safety Assessment

As shown in Table 1, all 42 candidate strains exhibited γ-hemolysis and were negative for mucin degradation, indicating that no evident hemolytic or mucin-degrading activity was detected under the conditions tested. Most isolates showed inhibition-zone diameters > 2.0 cm for all antibiotics examined under the present screening conditions. According to the predefined disk-diffusion criteria, inhibition-zone diameters of >2.0 cm, 1.5–2.0 cm, and ≤1.4 cm were classified as susceptible (S), intermediate (I), and resistant (R), respectively. Five isolates showed inhibition-zone diameters within the predefined intermediate screening range of 1.5–2.0 cm for one or more antibiotics. Specifically, *Lactiplantibacillus plantarum* MRS-25 and *Lactiplantibacillus pentosus* MC-14 and MC-17 showed intermediate susceptibility to amoxicillin (10 μg/disk), with inhibition-zone diameters falling within the intermediate range of 1.5–2.0 cm. *L. plantarum* M17-14 and *L. pentosus* MC-14 and MC-30 showed intermediate susceptibility to clindamycin (20 μg/disk), with inhibition-zone diameters also within the 1.5–2.0 cm range. MC-14 therefore exhibited intermediate susceptibility to both amoxicillin and clindamycin. No isolate showed an inhibition-zone diameter ≤ 1.4 cm under the present screening conditions. Because the present study adopted a conservative safety-first screening strategy, isolates showing intermediate susceptibility were excluded together with resistant isolates rather than being advanced to functional evaluation. Intermediate susceptibility was considered a potential safety signal because reduced susceptibility could not be distinguished from acquired resistance by disk diffusion alone and would require further confirmation by species-specific MIC determination and genomic analysis of antimicrobial-resistance determinants [20]. Accordingly, MRS-25, M17-14, MC-14, MC-17, and MC-30 were excluded, and the remaining 37 isolates were retained for subsequent evaluation of gastrointestinal tolerance, antagonistic activity, and adhesion-related properties.

### 3.4. In Vitro Functional Properties of the Candidate Strains

(1) Antagonistic Activity Against Indicator Pathogens

As summarized in Table 2 (the main text presents the nine selected strains, whereas the complete data for all 37 strains are provided in Table S1), all 37 candidate strains showed antagonistic activity against the three indicator pathogens, although the magnitude of inhibition differed among pathogens for the same strain. Sixteen strains—MRS-1, MRS-6, MRS-12, MRS-14, MRS-27, MRS-36, MRS-46, MRS-49, MRS-51, M17-5, M17-18, M17-24, M17-29, MC-10, MC-19, and MC-38—produced inhibition-zone widths > 0.6 cm against *E. coli*. Fourteen strains—MRS-1, MRS-6, MRS-12, MRS-16, MRS-19, MRS-27, MRS-36, MRS-46, MRS-49, MRS-51, M17-3, M17-24, M17-29, and MC-38—produced inhibition-zone widths > 1.0 cm against *S. dysenteriae*. Six strains—MRS-46, MRS-49, M17-2, M17-5, M17-24, and MC-24—produced inhibition-zone widths > 0.6 cm against *V. parahaemolyticus*. These results indicate selective antagonistic activity of sour bamboo shoot-derived LAB against different enteric pathogens. MRS-6, MRS-12, MRS-27, MRS-36, MRS-46, MRS-49, MRS-51, M17-24, and MC-38 showed relatively broad antagonistic activity, and most of these strains produced comparatively large inhibition zones against *S. dysenteriae*. Strain-to-strain variation indicates that pathogen antagonism is strain specific and cannot be predicted solely from taxonomic identity.

(2) Tolerance to Simulated Gastric and Intestinal Conditions

For LAB to remain viable during gastrointestinal transit, they must tolerate the acidic gastric environment, bile acids in the duodenum, and digestive enzymes. After 4 h of exposure to simulated gastric juice at pH 2.5, survival rates of the 37 strains ranged from 62.17% to 79.85% (Table 2), indicating a measurable level of gastric tolerance in all strains. After transfer to simulated intestinal conditions, however, differences among strains became more pronounced with increasing incubation time, showing that good gastric tolerance did not necessarily predict sustained intestinal tolerance. After 24 h in simulated intestinal fluid, survival declined markedly for most strains, whereas MRS-6, MRS-12, MRS-27, MRS-34, MRS-36, MRS-46, M17-22, M17-24, and MC-38 retained survival rates above 40%, indicating comparatively stronger persistence under the simulated gastrointestinal conditions.

(3) Adhesion-Related Phenotypes

Autoaggregation, coaggregation, and cell-surface hydrophobicity provide complementary indications of potential adhesion and interbacterial interaction. Most strains showed measurable autoaggregation, whereas MRS-13, M17-2, M17-4, and M17-5 exhibited autoaggregation values below 30%. Coaggregation was below 10% for M17-6 and M17-29, and cell-surface hydrophobicity was below 50% for MRS-14, MRS-16, M17-4, M17-36, and MC-5. These findings demonstrate that adhesion-related phenotypes were not concordant across strains; high performance in one trait did not necessarily correspond to favorable performance in the others. Integrating autoaggregation, coaggregation, and cell-surface hydrophobicity therefore provides a broader basis for identifying strains with favorable overall characteristics than selection based on a single adhesion-related trait.

### 3.5. PCA-Based Comprehensive Screening

PCA was performed using the eight in vitro functional variables obtained for the 37 candidate strains that passed the preliminary safety assessment. The KMO value was 0.786, and Bartlett’s test of sphericity was significant (df = 28, *p* < 0.001), indicating that the dataset was suitable for PCA. Two principal components with eigenvalues >1 were retained. PC1 and PC2 explained 38.73% and 14.16% of the variance, respectively (Table 3), accounting for 52.89% of the total variance. A comprehensive score was calculated using the variance contribution rates of the two components [F = (38.73 × F1 + 14.16 × F2)/52.89], and the strains were ranked accordingly. The nine highest-ranking strains, all with comprehensive scores > 0.7, were MRS-6, M17-24, MRS-12, MRS-34, MC-38, MRS-46, M17-22, MRS-27, and MRS-36 (Table 4). These strains were selected for subsequent in vitro fecal fermentation.

### 3.6. Effects of Candidate Strains on the Microbiota During In Vitro Fermentation of Patient-Derived Fecal Samples

#### 3.6.1. ASV Profiles Across Treatment Groups

**Figure 2:**
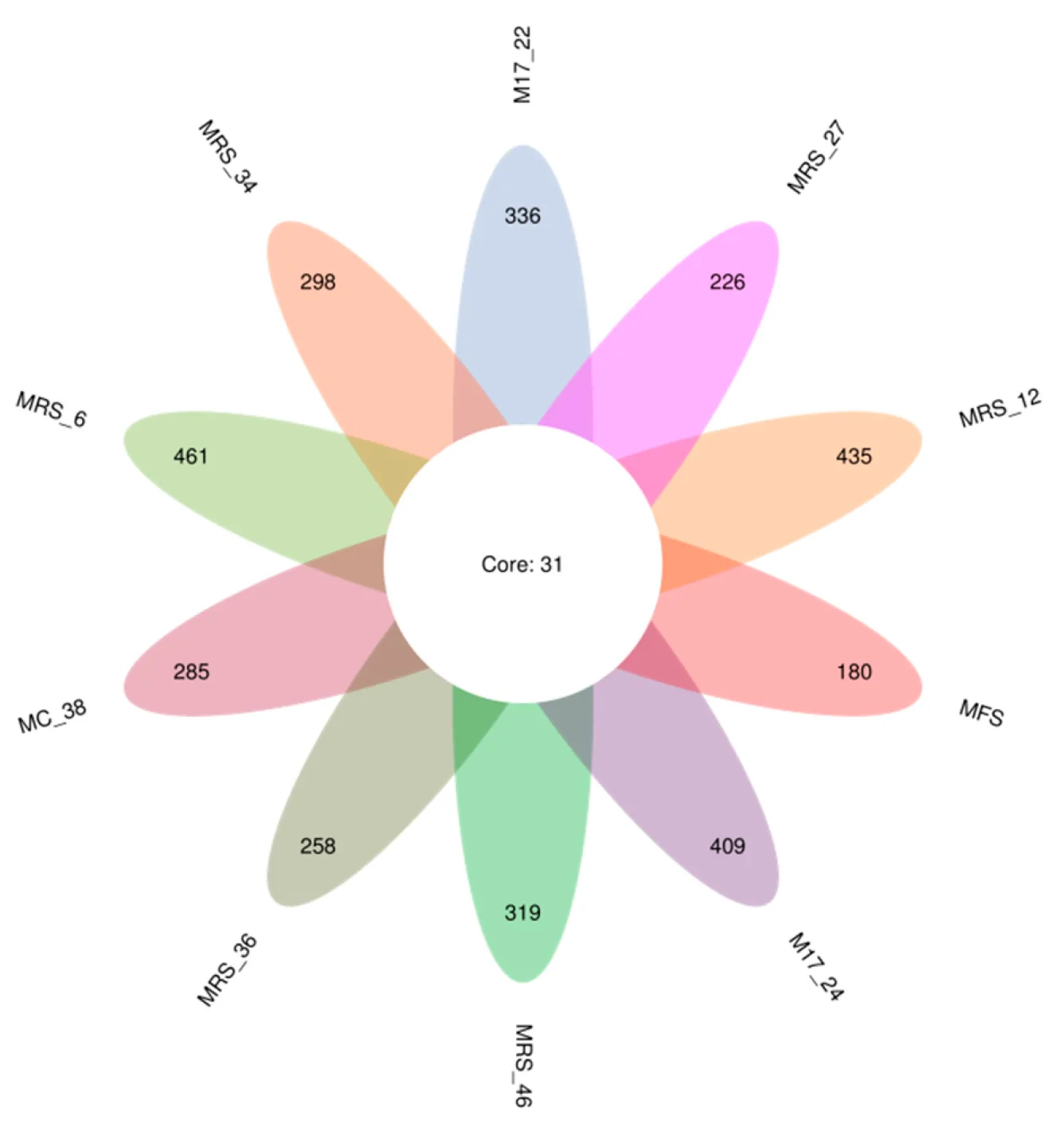
Flower plot summarizing the ASV composition across the different fermentation treatment groups. The central circle indicates the number of ASVs shared by all 10 groups (31 ASVs). Each petal represents one treatment group, and the number inside each petal indicates the number of unique ASVs in that group. The colors of the petals correspond to the different treatment groups as shown in the legend.

The flower plot (Figure 2) summarizes the ASV composition of the different treatment groups. Thirty-one ASVs were shared among all 10 fermentation groups, while each group also contained a variable number of unique ASVs, defined as distinct denoised amplicon sequences. Compared with the control without an added candidate strain, some strain-treated samples contained more unique ASVs, suggesting that candidate-strain supplementation affected not only the relative composition of dominant taxa but also the broader community structure within the in vitro fermentation system. The distinct ASV patterns among treatments indicate strain-specific responses in the complex fecal microbial community.

#### 3.6.2. Alpha Diversity of Bacterial Communities

**Figure 3:**
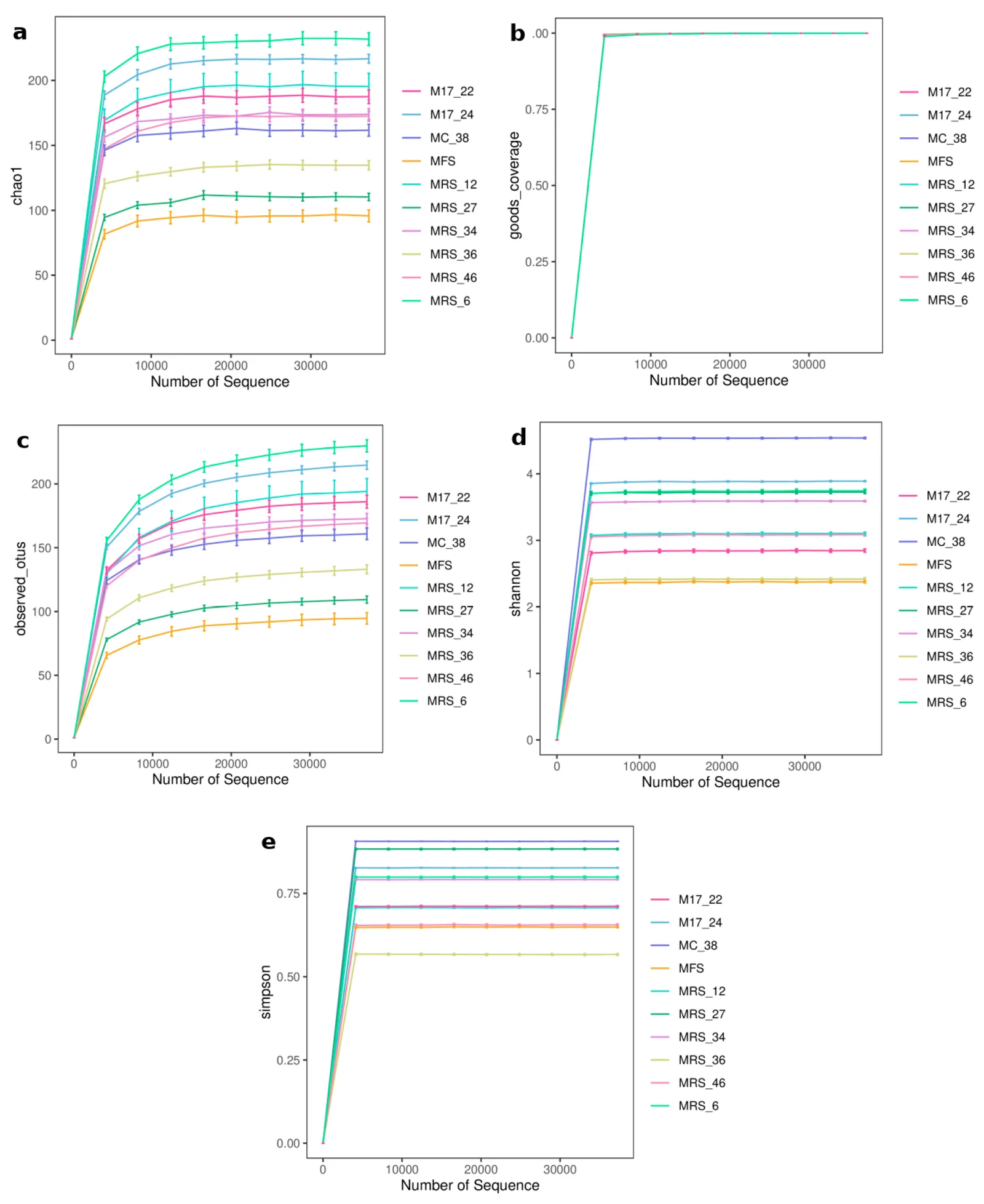
Rarefaction curves of alpha-diversity metrics for bacterial communities across treatment groups: (**a**) Chao1; (**b**) Good’s coverage; (**c**) observed ASVs; (**d**) Shannon index; and (**e**) Simpson index.

Rarefaction curves approached saturation across the treatment groups, and Good’s coverage values ranged from 0.998 to 1.000, indicating that the sequencing depth was sufficient to capture the major bacterial populations in the in vitro fermentation samples (Figure 3). The rarefaction curves were therefore interpreted primarily as an assessment of sequencing completeness rather than as evidence of between-group differences in alpha diversity. Chao1, observed ASVs, Shannon, and Simpson indices were calculated independently for each fermentation replicate and were subjected to formal statistical comparison among treatment groups using the Kruskal–Wallis test, followed, where appropriate, by Dunn’s pairwise comparisons with Benjamini–Hochberg FDR correction. No significant overall differences were detected among treatment groups for Chao1, observed ASVs, Shannon, or Simpson indices after FDR-adjusted comparisons (*q* > 0.05). Overall, these results indicate that the candidate LAB did not significantly alter microbial richness or alpha diversity under the present in vitro fermentation conditions.

#### 3.6.3. Beta Diversity of Bacterial Communities

**Figure 4:**
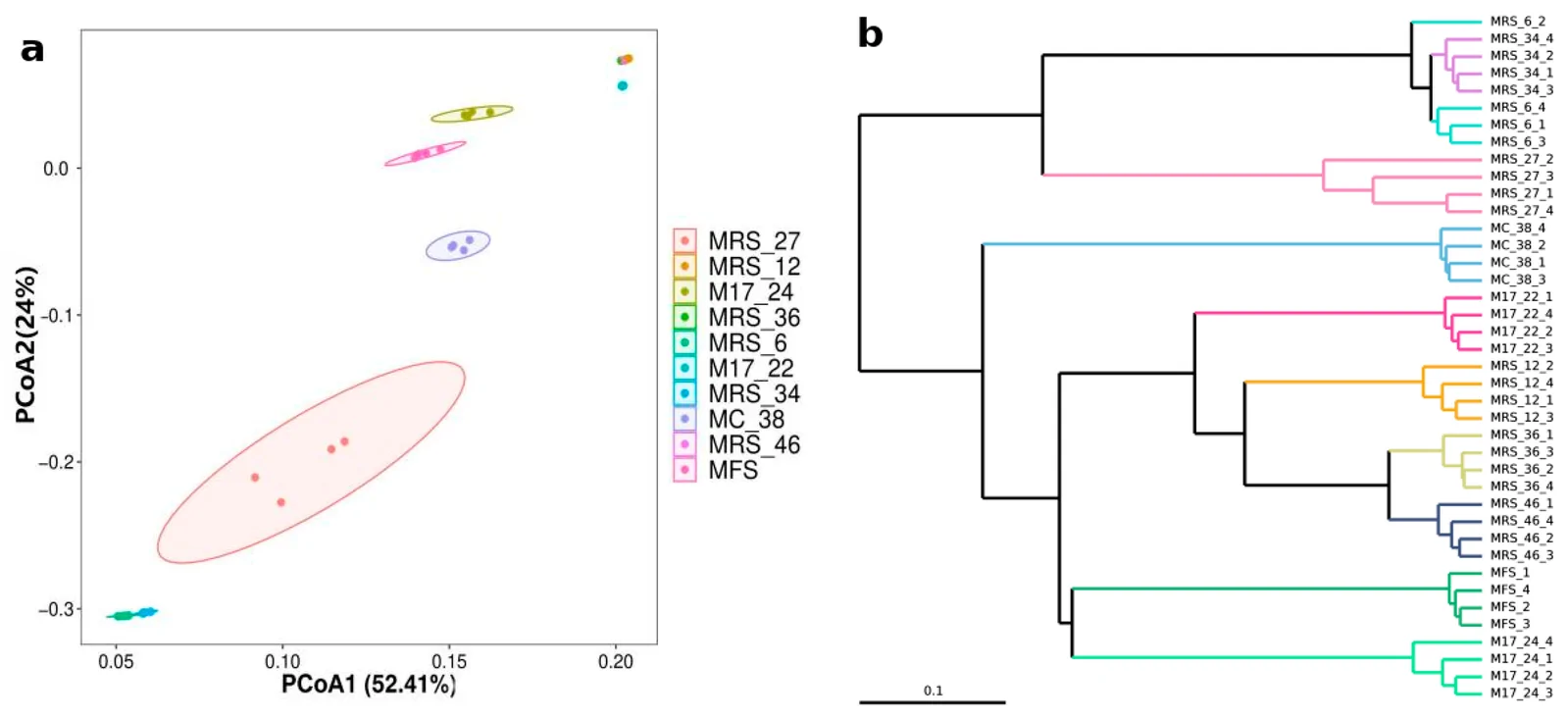
Principal coordinates analysis (PCoA) and hierarchical clustering of bacterial communities across treatment groups: (**a**) PCoA; (**b**) hierarchical clustering. Colors indicate different treatment groups.

β-diversity analysis based on Bray–Curtis dissimilarity revealed differences in bacterial community composition among the treatment groups. As shown in Figure 4a, PCoA1 and PCoA2 explained 52.41% and 24.00% of the total variation, respectively, accounting for 76.41% of the variation in community composition. The M17-24 group was positioned relatively close to the MFS control, whereas several other LAB-treated groups showed greater separation from the control. PERMANOVA based on the Bray–Curtis distance matrix further demonstrated significant differences in bacterial community composition among the treatment groups (pseudo-F = 7.93, R^2^ = 0.704, *p* = 0.001), indicating that treatment explained approximately 70.4% of the overall variation in microbial community structure. Hierarchical clustering showed a pattern broadly consistent with the PCoA ordination, with replicates within individual treatments clustering closely and the M17-24 and MFS groups showing relatively high similarity. Collectively, these results indicate that the candidate LAB exerted strain-specific effects on the overall structure of the patient-derived fecal microbial community.

#### 3.6.4. Bacterial Community Composition Across Treatment Groups

**Figure 5:**
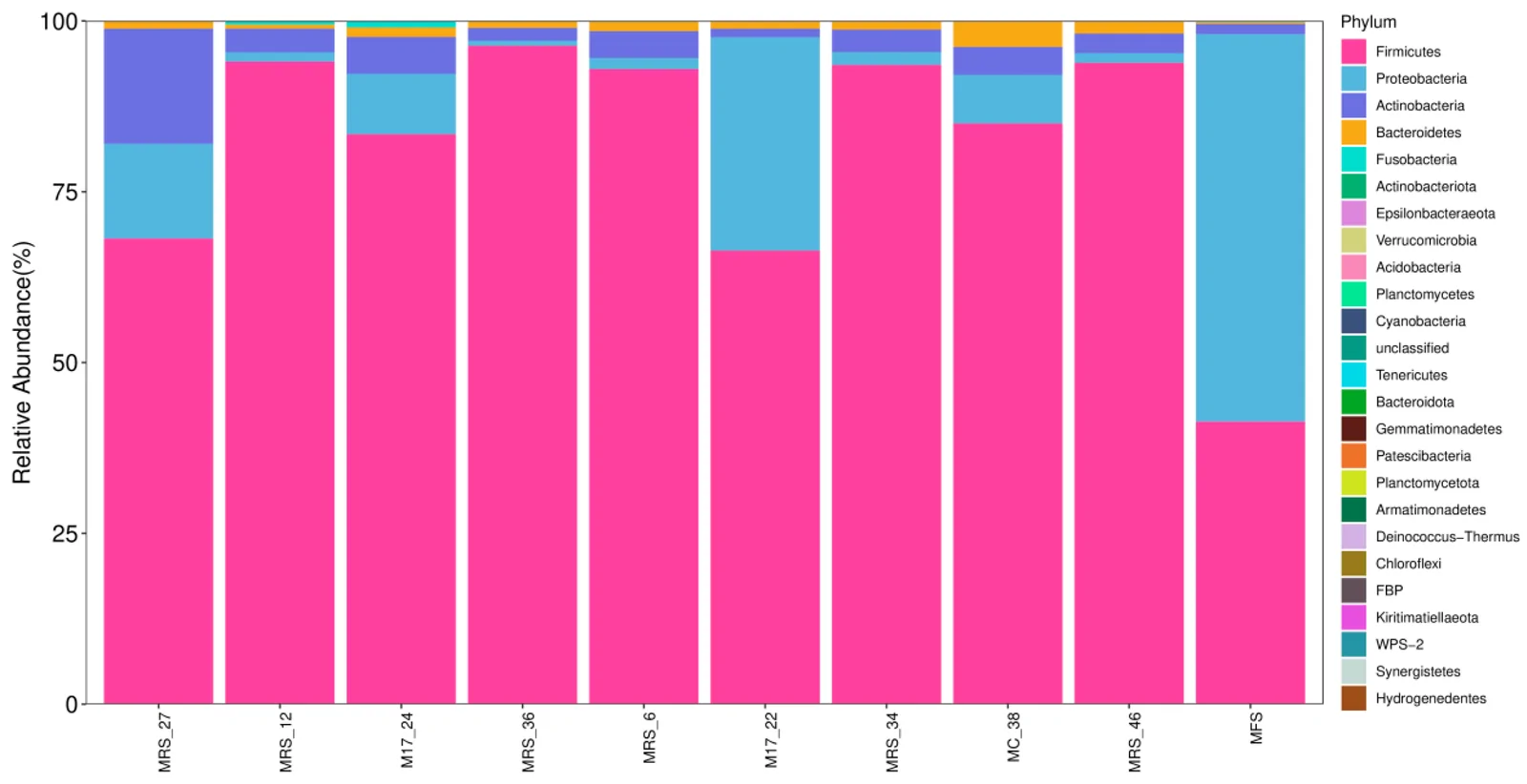
Relative abundance of bacterial taxa at the phylum level across treatment groups.

At the phylum level (Figure 5), the bacterial communities were dominated by Firmicutes, Proteobacteria, Actinobacteria, and Bacteroidetes. In the MFS control after fecal fermentation, Firmicutes, Proteobacteria, and Actinobacteria accounted for 41.37%, 56.70%, and 1.49% of the community, respectively. After supplementation with MRS-6, MRS-12, MRS-27, MRS-34, MRS-36, MRS-46, M17-22, M17-24, or MC-38 and subsequent in vitro fermentation, the relative abundances of Firmicutes and Bacteroidetes increased, whereas that of Proteobacteria decreased. These shifts indicate that the candidate LAB altered the relative proportions of dominant bacterial phyla during in vitro fermentation, although the magnitude of the response differed among strains.

**Figure 6:**
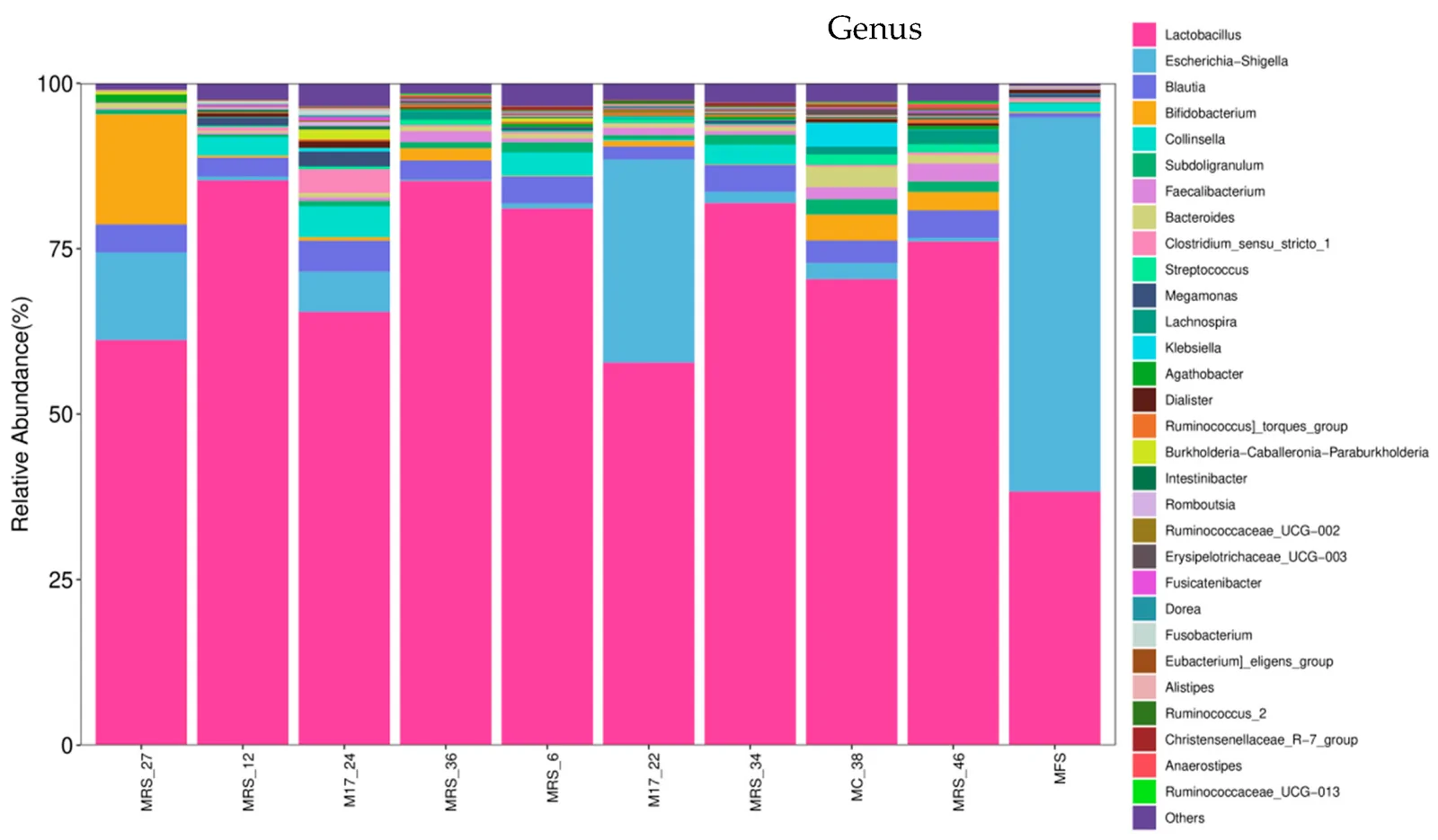
Relative abundance of bacterial taxa at the genus level across treatment groups.

At the genus level (Figure 6), *Escherichia–Shigella* and *Lactobacillus* dominated the MFS control, accounting for 56.55% and 38.29% of the community, respectively. Following 48 h of fermentation with the candidate LAB, the relative abundance of *Escherichia–Shigella* decreased to varying degrees in all treatment groups, indicating an association between candidate-strain supplementation and reduced *Escherichia–Shigella* relative abundance. *Escherichia–Shigella* decreased to below 1% in the MRS-6, MRS-12, MRS-36, and MRS-46 groups and to below 3% in the MRS-34 and MC-38 groups. The relative abundance was 6.09% in the M17-24 group, 13.26% in the MRS-27 group, and 30.71% in the M17-22 group, indicating a comparatively weaker response to M17-22. At the same time, the relative abundances of commensal genera including *Blautia*, *Bifidobacterium*, *Collinsella*, *Faecalibacterium*, and *Bacteroides* increased to varying extents. Thus, candidate-strain supplementation was associated not only with a reduction in *Escherichia–Shigella* relative abundance but also with concurrent changes in several health-associated commensal taxa, suggesting broader restructuring of the microbial community.

**Figure 7:**
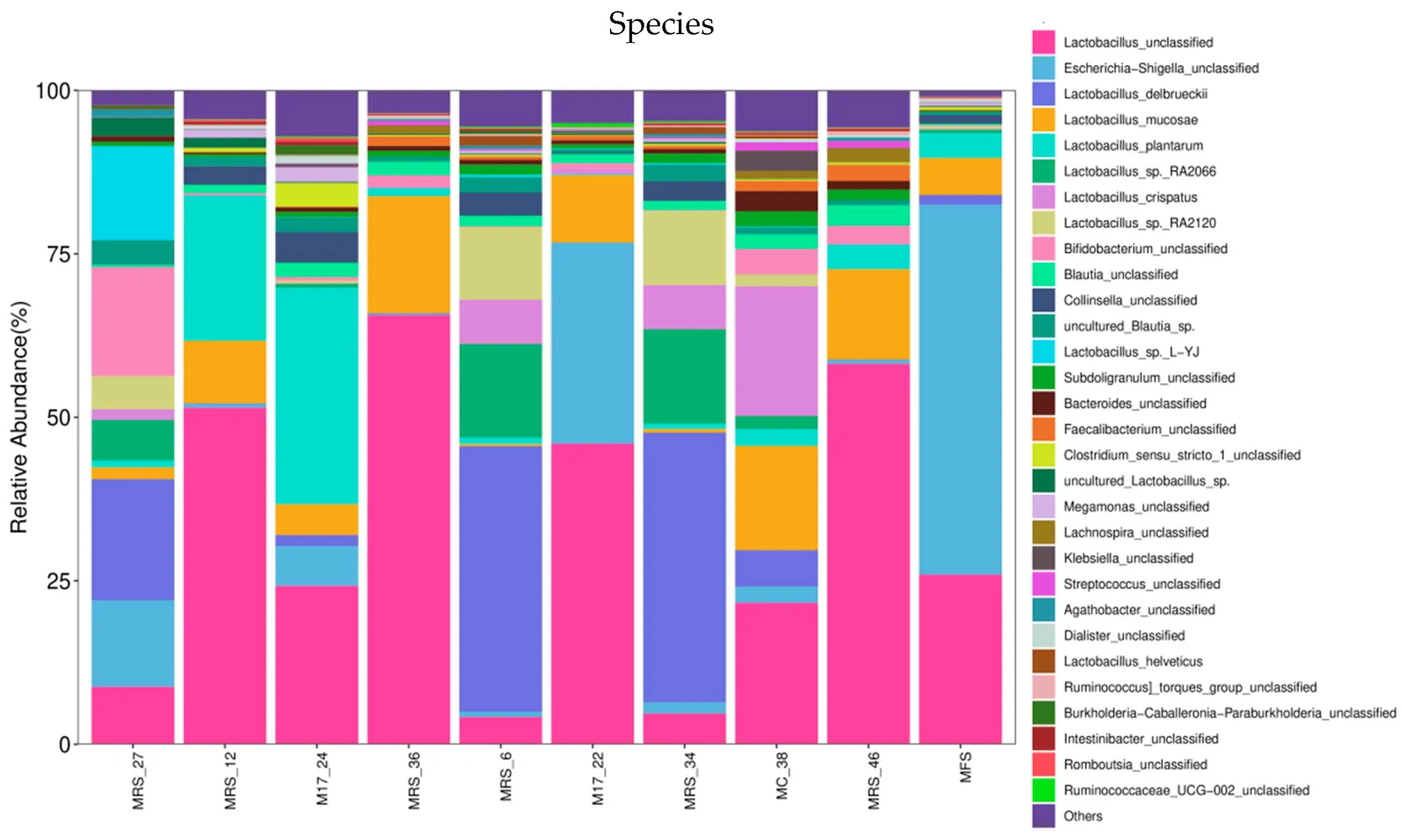
Relative abundance of bacterial taxa assigned at the species level across treatment groups.

At the species-assignment level (Figure 7), the distribution of *Escherichia–Shigella* across treatments was consistent with the genus-level pattern, with the M17-22 group retaining a comparatively high relative abundance. Other treatment groups contained relatively high proportions of lactobacilli. Taxa assigned as *Lactobacillus delbrueckii* accounted for 40.61%, 41.23%, 18.49%, and 5.55% in the MRS-6, MRS-34, MRS-27, and MC-38 groups, respectively. MRS-6 and MRS-34 also contained relatively high proportions of taxa assigned as *Lactobacillus* sp. RA2066 (14.32% and 14.45%, respectively). Taxa assigned as *Limosilactobacillus mucosae* were relatively abundant in the MRS-36, MC-38, MRS-46, M17-22, and MRS-12 groups. Taxa assigned as *Lactiplantibacillus plantarum* accounted for 33.12% and 22.24% in the M17-24 and MRS-12 groups, respectively, whereas *Bifidobacterium* accounted for 16.65% in the MRS-27 group. These differences indicate that the candidate strains were associated with distinct patterns not only in the target taxon but also among lactobacilli and other commensal bacteria, resulting in strain-specific community responses.

**Figure 8:**
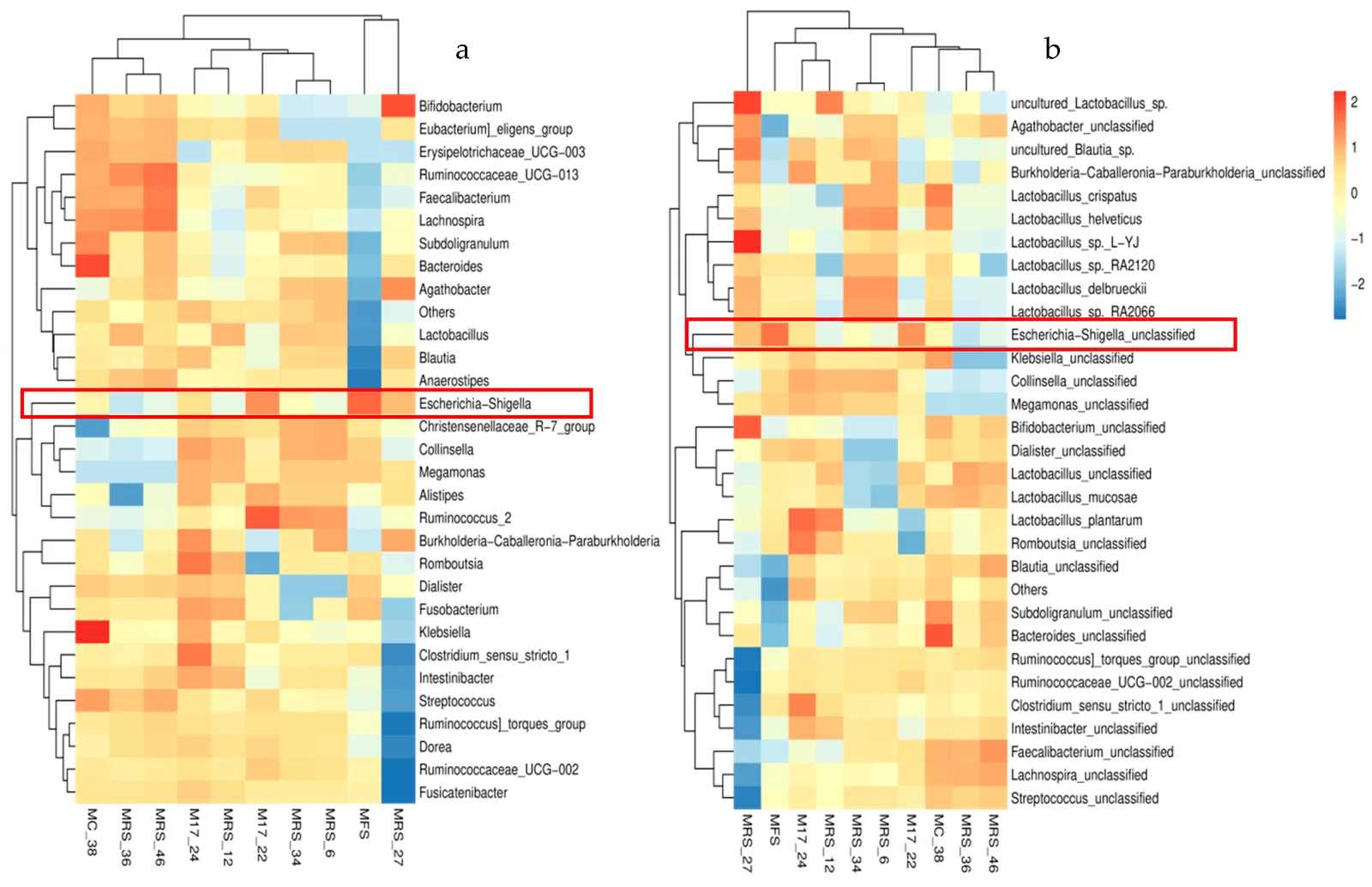
Hierarchical clustering heatmap of bacterial taxa across treatment groups: (**a**) genus-level assignments; (**b**) species-level assignments. The red box highlights the relative abundance of *Escherichia-Shigella*.

The hierarchical clustering heatmap (Figure 8) was used to visualize treatment-associated patterns in bacterial relative abundance, whereas statistical comparisons were performed separately using replicate-level relative-abundance data. At the genus level, the Kruskal–Wallis test revealed a significant overall treatment effect on the relative abundance of *Escherichia–Shigella* (H = 32.86, *p* < 0.001). Subsequent Dunn’s pairwise comparisons with Benjamini–Hochberg FDR correction showed that *Escherichia–Shigella* relative abundance was significantly lower in the MRS-6 (*q* = 0.003), MRS-12 (*q* = 0.004), MRS-34 (*q* = 0.011), MRS-36 (*q* = 0.002), MRS-46 (*q* = 0.002), MC-38 (*q* = 0.014), and M17-24 (q = 0.028) groups than in the MFS control. The reduction in the MRS-27 group showed a weaker but statistically significant difference (*q* = 0.043), whereas the difference in the M17-22 group was not significant after FDR correction (*q* = 0.176). *Lactobacillus* relative abundance also differed significantly among treatment groups (Kruskal–Wallis, H = 27.41, *p* = 0.001). Dunn’s post hoc analysis indicated significantly higher relative abundances in the MRS-6 (*q* = 0.012), MRS-12 (*q* = 0.018), MRS-34 (q = 0.009), MRS-36 (*q* = 0.021), and MRS-46 (*q* = 0.016) groups compared with the MFS control, whereas the remaining comparisons did not remain significant after FDR correction (*q* > 0.05). Overall, these results indicate that selected candidate LAB were associated with statistically supported, strain-dependent changes in specific bacterial taxa, particularly *Escherichia–Shigella*.

### 3.7. Effects of Candidate LAB on SCFAs During In Vitro Fermentation of Patient-Derived Fecal Samples

In this study, the 10% fecal fermentation suspension before and after fermentation served as common reference points, and SCFA concentrations after 48 h were compared among the MFS control and the nine candidate-strain treatment groups. As shown in Table 5, total SCFAs in the MFS group decreased from 9.35 mmol/L before fermentation to 7.02 mmol/L after 48 h, with acetate decreasing from 5.81 to 3.52 mmol/L and butyrate from 1.21 to 0.43 mmol/L. This pattern indicates substantial changes in SCFA production and consumption during fermentation in the absence of an added candidate strain. SCFA profiles differed markedly among strain treatments. Total SCFAs reached 14.51, 13.55, and 12.16 mmol/L in the MRS-36, MRS-46, and MRS-6 groups, respectively, and were also relatively high in the MRS-12 (10.94 mmol/L), MC-38 (10.52 mmol/L), and MRS-34 (10.42 mmol/L) groups. By contrast, total SCFAs were 7.18 mmol/L in the MRS-27 group, 8.32 mmol/L in the M17-24 group, and only 5.02 mmol/L in the M17-22 group, demonstrating pronounced strain-specific metabolic effects. The largest differences among individual SCFAs were observed for acetate, propionate, and butyrate. Acetate and butyrate reached 7.85 and 3.25 mmol/L, respectively, in the MRS-36 group and 7.45 and 2.77 mmol/L in the MRS-46 group. Notably, butyrate in the MFS group after fermentation was only 0.43 mmol/L, whereas all candidate-strain groups except M17-22 showed significantly higher butyrate concentrations (*p* < 0.05). Butyrate concentrations in the MRS-36, MRS-46, MC-38, MRS-12, and MRS-34 groups were 3.25, 2.77, 2.34, 2.23, and 2.04 mmol/L, respectively. Notably, the treatment groups showing relatively high total SCFA concentrations, particularly MRS-36, MRS-46, and MRS-6, also exhibited relatively low abundances of *Escherichia–Shigella* in the 16S rRNA gene sequencing analysis. However, the present study did not perform a direct correlation analysis between SCFA concentrations and *Escherichia–Shigella* relative abundance, and therefore a causal relationship between SCFA accumulation and the reduction in *Escherichia–Shigella* cannot be established. The SCFA data were included as a complementary metabolic endpoint to the microbiota analysis, reflecting strain-dependent changes in microbial metabolic activity during in vitro fermentation rather than a direct mechanism of *Escherichia–Shigella* inhibition.

## 4. Discussion

Lactobacilli are common members of the human intestinal microbiota and are also important in fermented-food production [21]. Strains belonging to lactobacilli, bifidobacteria, and lactococci with favorable probiotic properties have been widely used commercially [22], and the probiotic functions of lactobacilli remain an active area of research [23]. Naturally fermented foods are important sources of LAB with potential functional properties. In the present study, LAB isolated from sour bamboo shoots included *Lactiplantibacillus plantarum*, *L. pentosus*, *Limosilactobacillus fermentum*, *Weissella confusa*, and *Pediococcus pentosaceus*. Previous studies have identified *Lactobacillus*, *Weissella*, and *Pediococcus* as important members of the microbial communities in sour bamboo shoots and other fermented vegetables [24,25], supporting the view that naturally fermented sour bamboo shoots harbor a diverse LAB reservoir. Probiotic strains must undergo safety assessment before being used as food supplements [26,27]. None of the 42 candidate strains showed hemolytic or mucin-degrading activity, whereas five strains were excluded because of intermediate antibiotic resistance. Even LAB derived from traditional fermented foods require evaluation of antibiotic resistance, hemolysis, and potential virulence risks [28,29]. The present safety-first, function-second strategy was therefore appropriate and yielded LAB from multiple genera and species, consistent with the reported diversity of sour bamboo shoot fermentation microbiota. Previous studies have isolated *L. plantarum* strains from bamboo shoot fermentation systems with favorable gastrointestinal tolerance, adhesion, and antagonistic activity against pathogens [6]. LAB must withstand gastric acid, bile salts, and digestive enzymes to survive gastrointestinal transit [30,31], and such tolerance varies markedly among strains [32]. Therefore, gastrointestinal tolerance should be considered an important criterion for candidate strain selection. Nevertheless, the present safety assessment was limited to preliminary phenotypic screening. For strains considered for further development, particularly MRS-36, MIC testing and whole-genome sequencing will be required to determine whether transferable antimicrobial-resistance genes or virulence factors are present [29].

The 37 strains that passed the safety assessment differed markedly in gastrointestinal tolerance, pathogen antagonism, and adhesion-related phenotypes, highlighting the strain specificity of putative probiotic traits. Similar variation has been reported among LAB isolated from bamboo shoots and other fermented foods [6,28]. PCA was subsequently used to integrate eight functional variables and identify nine selected strains. Multivariate approaches can reduce the bias introduced by selection based on a single functional trait and have been used in other LAB screening studies [6,33]. Importantly, the PCA ranking did not fully predict strain behavior in the complex fecal community. M17-24 ranked highly by PCA but produced relatively limited changes in microbiota composition and SCFAs, whereas MRS-36 ranked lower by PCA yet showed a stronger microecological response. These findings indicate that functional assays under pure-culture conditions are most useful for preliminary candidate screening and should be complemented by validation in complex microbial communities.

In vitro fecal fermentation showed that individual LAB strains altered microbial community composition to different extents. The relative abundance of *Escherichia–Shigella* decreased to below 1% after treatment with MRS-6, MRS-12, MRS-36, or MRS-46, while commensal genera including *Blautia*, *Bifidobacterium*, *Faecalibacterium*, and *Bacteroides* increased to varying degrees. Previous in vitro fecal fermentation and human intervention studies have likewise reported reductions in potentially pathogen-associated taxa such as *Escherichia–Shigella*, together with increases in selected health-associated commensals following treatment with LAB or their metabolites [34,35,36]. However, 16S rRNA gene sequencing measures relative rather than absolute abundance; therefore, the present data support an association between candidate-strain treatment and lower *Escherichia–Shigella* relative abundance but do not establish an absolute reduction in cell numbers. A human intervention study also reported a decrease in *Escherichia–Shigella* after consumption of fermented milk containing *L. paracasei*, accompanied by changes in health-associated taxa and SCFAs [37]. Thus, the *Escherichia–Shigella* pattern observed here is consistent with a broader body of evidence linking LAB interventions to compositional shifts in complex intestinal microbial communities.

SCFAs are major metabolites produced by microbial fermentation in the gut, and changes in their concentrations provide a metabolic readout of functional responses in in vitro microbial communities [38,39,40]. Specific LAB interventions have been associated with reduced *Escherichia–Shigella* relative abundance and increased acetate, propionate, and butyrate concentrations, and fermented bamboo shoot products have produced similar microbiota and SCFA responses in a colitis animal model [41].In this study, total SCFA concentrations were higher in the MRS-36, MRS-46, and MRS-6 groups, with the most pronounced accumulation of acetate and butyrate observed in the MRS-36 and MRS-46 groups. This pattern was broadly consistent with the findings of Ohashi [10]. In contrast, changes in isobutyrate, isovalerate, valerate, and hexanoate did not fully parallel total SCFAs, indicating that the increase in total SCFAs was driven mainly by acetate, propionate, and butyrate and exhibited clear strain-specific differences. Together with the 16S rRNA gene sequencing results, these metabolic changes may be associated with microbial cross-feeding; for example, acetate and lactate produced by bifidobacteria can be further utilized by *Faecalibacterium*, *Eubacterium*, and other taxa for butyrate production, a phenomenon also reported in patient-derived in vitro microbial community models [42]. However, because metabolic pathways and correlation analyses were not examined in the present study, these findings should be interpreted as concurrent changes in microbial community composition and SCFA profiles rather than evidence of a direct causal relationship.

Several limitations should be acknowledged. Fecal samples from the five patients were pooled in equal proportions, generating a single composite microbial background and precluding assessment of donor-specific responses to the candidate strains. In addition, V3–V4 amplicon sequencing has limited species-level resolution for closely related taxa, and the in vitro fermentation system does not reproduce host epithelial, immune, or absorptive processes. Future studies should therefore use independent donor-level fermentation replicates and incorporate qPCR, whole-genome sequencing, metagenomics, and/or metabolomics for further validation. Overall, the present study established a sequential workflow comprising preliminary safety screening, multivariate functional evaluation, PCA-based selection, and validation in a complex fecal microbial community using both microbiota and SCFA endpoints. Among the selected strains, MRS-36 and MRS-46 showed the most consistent microbiota and metabolic responses and should be prioritized for further investigation.

## 5. Conclusions

This study identified a set of sour bamboo shoot-derived LAB with potential functional value and, more importantly, established a sequential screening strategy integrating preliminary safety exclusion, multivariate functional evaluation, PCA-based selection, and validation in a complex microbial community. By extending strain-level phenotypic screening to functional evaluation in an in vitro fecal microbial community, this approach addresses some of the limitations of selecting candidate probiotic strains solely on the basis of individual acid-tolerance, antimicrobial, or adhesion-related traits. The results further show that favorable performance in conventional in vitro assays does not necessarily translate into an equivalent response within a complex microbial community. Accordingly, combining multivariate screening with in vitro fecal fermentation may improve the specificity of candidate-strain prioritization. These findings expand current knowledge of the functional diversity of LAB from traditionally fermented sour bamboo shoots and provide a strain and methodological basis for subsequent whole-genome safety assessment, metabolic characterization, mechanistic studies, and development of microbiota-oriented functional preparations.

## Figures and Tables

**Table 1:** Preliminary phenotypic safety assessment of the candidate strains.

Strain	MRS-25	M17-14	MC-14	MC-17	MC-30	Other
Amoxicillin (10 µg/disk)	R	S	I	I	S	S
Ampicillin (10 µg/disk)	S	S	S	S	S	S
Levofloxacin (10 µg/disk)	S	S	S	S	S	S
Chloramphenicol (30 µg/disk)	S	S	S	S	S	S
Erythromycin (15 µg/disk)	S	S	S	S	S	S
Clindamycin (20 µg/disk)	S	I	I	S	R	S
Tetracycline (30 µg/disk)	S	S	S	S	S	S
Vancomycin (30 µg/disk)	S	S	S	S	S	S
Cefoperazone (20 µg/disk)	S	S	S	S	S	S
Gentamicin (10 µg/disk)	S	S	S	S	S	S
Tobramycin (10 µg/disk)	S	S	S	S	S	S
Streptomycin (300 µg/disk)	S	S	S	S	S	S
Hemolytic activity	γ	γ	γ	γ	γ	γ
Mucin degradation	Neg.	Neg.	Neg.	Neg.	Neg.	Neg.

Note: “Other” includes MRS-1, MRS-6, MRS-12, MRS-13, MRS-14, MRS-16, MRS-18, MRS-19, MRS-22, MRS-27, MRS-34, MRS-36, MRS-46, MRS-49, MRS-51, M17-2, M17-3, M17-4, M17-5, M17-6, M17-7, M17-8, M17-10, M17-18, M17-22, M17-24, M17-29, M17-30, M17-36, M17-38, MC-1, MC-4, MC-5, MC-10, MC-19, MC-24, and MC-38, which showed identical results. Neg., negative. The categories S (diameter > 2.0 cm), I (2.0 cm ≥ diameter > 1.4 cm), and R (diameter ≤ 1.4 cm) in this table are used for preliminary phenotypic screening and should not be considered as CLSI clinical breakpoints for specific species or specific antimicrobial agents. S, susceptible; I, intermediate; R, resistant; Neg., negative; CLSI, Clinical and Laboratory Standards Institute. γ-hemolysis indicates the absence of hemolytic activity.

**Table 2:** In vitro physiological and functional properties of the nine selected strains.

Strain	Antagonistic Activity: Inhibition-Zone Width (cm)			Gastrointestinal Tolerance: Survival Rate (%)		Adhesion-Related Properties (%)		
	Ec-AA	Sd-AA	Vp-AA	GJ-SR	SIF-SR	Auto-C	Co-C	CSH
MRS-6	0.83 ± 0.04	1.53 ± 0.13	0.55 ± 0.08	79.33 ± 1.06	51.28 ± 0.38	45.12 ± 1.87	17.65 ± 0.80	78.77 ± 5.46
MRS-12	0.75 ± 0.26	1.55 ± 0.09	0.48 ± 0.05	78.43 ± 1.21	48.28 ± 1.72	43.33 ± 2.39	23.32 ± 1.11	85.33 ± 2.54
MRS-27	0.70 ± 0.07	1.33 ± 0.04	0.24 ± 0.06	77.67 ± 1.16	50.32 ± 1.65	50.34 ± 0.45	24.76 ± 1.68	83.62 ± 4.67
MRS-34	0.73 ± 0.06	1.33 ± 0.06	0.62 ± 0.02	77.42 ± 1.22	50.30 ± 1.48	46.47 ± 1.06	24.02 ± 1.03	78.35 ± 4.29
MRS-36	0.68 ± 0.07	1.60 ± 0.20	0.40 ± 0.07	77.75 ± 0.08	40.41 ± 1.40	39.22 ± 3.65	28.76 ± 1.56	67.85 ± 3.61
MRS-46	0.61 ± 0.04	1.53 ± 0.15	0.64 ± 0.08	76.08 ± 1.20	46.21 ± 0.66	54.12 ± 1.40	26.76 ± 1.12	69.46 ± 1.40
M17-22	0.83 ± 0.03	1.13 ± 0.20	0.53 ± 0.08	79.21 ± 1.36	42.91 ± 1.90	49.13 ± 3.40	16.35 ± 1.14	64.27 ± 3.10
M17-24	0.73 ± 0.15	1.58 ± 0.05	0.67 ± 0.05	76.40 ± 1.22	43.42 ± 1.20	48.34 ± 2.50	16.80 ± 0.70	79.06 ± 0.73
MC-38	0.70 ± 0.16	1.52 ± 0.11	0.53 ± 0.02	75.93 ± 0.96	40.05 ± 2.01	41.33 ± 2.61	24.28 ± 1.38	86.75 ± 6.28

Note: Values are expressed as mean ± SD. Ec-AA, antagonistic activity against *Escherichia coli*, expressed as inhibition-zone width; Sd-AA, antagonistic activity against Shigella dysenteriae, expressed as inhibition-zone width; Vp-AA, antagonistic activity against Vibrio parahaemolyticus, expressed as inhibition-zone width; GJ-SR, survival rate in simulated gastric juice (pH 2.5, 4 h); SIF-SR, survival rate in simulated intestinal fluid (24 h); Auto-C, autoaggregation capacity; Co-C, coaggregation capacity with *E. coli*; CSH, cell-surface hydrophobicity. Antagonistic activity is expressed in cm, whereas survival rates, aggregation capacities, and cell-surface hydrophobicity are expressed as percentages.

**Table 3:** Total variance explained, component loadings, and score coefficients for the first two principal components.

PC	Eigenvalue	Variance (%)	Cumulative (%)	Variable	PC1 Loading	PC2 Loading	PC1 Score Coeff.	PC2 Score Coeff.
PC1	3.10	38.73	38.73	Ec-AA	0.784	0.051	0.253	0.045
PC2	1.13	14.16	52.89	Sd-AA	0.769	−0.263	0.248	−0.232
PC3	0.94	11.79	64.68	Vp-AA	0.436	0.604	0.141	0.533
PC4	0.87	10.93	75.61	GJ-SR	0.686	0.203	0.221	0.179
PC5	0.63	7.86	83.48	SIF-SR	0.725	−0.062	0.234	−0.055
PC6	0.55	6.82	90.30	Auto-C	0.600	−0.514	0.194	−0.454
PC7	0.39	4.93	95.22	Co-C	0.406	−0.297	0.131	−0.262
PC8	0.38	4.78	100	CSH	0.428	0.547	0.138	0.483

**Table 4:** The nine highest-ranking candidate lactic acid bacteria based on principal component analysis (PCA)-derived comprehensive scores.

No.	Strain	PC1 Score	PC2 Score	Comprehensive Score (F)
1	MRS-6	1.92	0.51	1.54
2	M17-24	1.67	0.65	1.40
3	MRS-12	1.80	0.23	1.38
4	MRS-34	1.76	0.33	1.38
5	MC-38	1.46	0.48	1.19
6	MRS-46	1.75	−0.77	1.08
7	M17-22	1.38	0.03	1.01
8	MRS-27	1.54	−1.13	0.82
9	MRS-36	1.32	−0.80	0.75

Note: The remaining strains (*n* = 28) had comprehensive scores < 0.7 and were not ranked. PC, principal component; F, comprehensive score.

**Table 5:** Effects of candidate lactic acid bacteria on short-chain fatty acid concentrations in the in vitro fecal fermentation system.

Group	SCFAs (mmol/L)							Total
	Acetate	Propionate	i-Butyrate	Butyrate	i-Valerate	Valerate	Hexanoate	
MFS (before)	5.81 ± 0.38 ^c^	1.24 ± 0.08 ^e^	0.38 ± 0.04 ^cd^	1.21 ± 0.09 ^e^	0.18 ± 0.01 ^d^	0.25 ± 0.03 ^d^	0.28 ± 0.04 ^bc^	9.35
MFS (after)	3.52 ± 0.45 ^e^	1.35 ± 0.10 ^de^	0.45 ± 0.04 ^b^	0.43 ± 0.04 ^g^	0.36 ± 0.02 ^a^	0.50 ± 0.03 ^a^	0.41 ± 0.05 ^a^	7.02
MRS-6	7.23 ± 0.66 ^a^	1.95 ± 0.14 ^bc^	0.31 ± 0.06 ^d^	1.89 ± 0.11 ^d^	0.31 ± 0.04 ^ab^	0.41 ± 0.04 ^b^	0.06 ± 0.01 ^e^	12.16
MRS-12	6.08 ± 0.45 ^b^	1.67 ± 0.14 ^c^	0.34 ± 0.03 ^d^	2.23 ± 0.15 ^c^	0.24 ± 0.01 ^c^	0.32 ± 0.06 ^c^	0.06 ± 0.01 ^e^	10.94
MRS-27	3.92 ± 0.26 ^de^	1.34 ± 0.16 ^de^	0.42 ± 0.03 ^c^	0.88 ± 0.01 ^f^	0.13 ± 0.04 ^de^	0.35 ± 0.03 ^c^	0.14 ± 0.01 ^d^	7.18
MRS-34	5.64 ± 0.11 ^c^	1.52 ± 0.09 ^c^	0.46 ± 0.05 ^b^	2.04 ± 0.14 ^d^	0.20 ± 0.06 ^c^	0.46 ± 0.07 ^a^	0.10 ± 0.02 ^d^	10.42
MRS-36	7.85 ± 0.64 ^a^	2.12 ± 0.11 ^b^	0.56 ± 0.06 ^a^	3.25 ± 0.25 ^a^	0.24 ± 0.01 ^c^	0.14 ± 0.01 ^e^	0.35 ± 0.04 ^ab^	14.51
MRS-46	7.45 ± 0.55 ^a^	2.55 ± 0.12 ^a^	0.24 ± 0.01 ^e^	2.77 ± 0.41 ^b^	0.26 ± 0.02 ^bc^	0.16 ± 0.02 ^e^	0.12 ± 0.01 ^d^	13.55
M17-22	1.85 ± 0.11 ^f^	1.67 ± 0.12 ^c^	0.29 ± 0.01 ^d^	0.45 ± 0.02 ^g^	0.06 ± 0.01 ^f^	0.45 ± 0.04 ^ab^	0.25 ± 0.06 ^c^	5.02
M17-24	4.16 ± 0.32 ^d^	1.25 ± 0.13 ^e^	0.45 ± 0.07 ^b^	0.85 ± 0.03 ^f^	0.09 ± 0.01 ^e^	0.21 ± 0.01 ^d^	0.31 ± 0.02 ^b^	8.32
MC-38	5.46 ± 0.37 ^c^	1.47 ± 0.20 ^cd^	0.46 ± 0.04 ^b^	2.34 ± 0.22 ^c^	0.12 ± 0.02 ^e^	0.26 ± 0.02 ^d^	0.41 ± 0.05 ^a^	10.52

Note: Different superscript letters within the same column indicate significant differences (*p* < 0.05).

## Data Availability

The original contributions presented in this study are included in the article and Supplementary Material. Further inquiries can be directed to the corresponding author.

## Supplementary Materials

The following supporting information can be downloaded at: https://www.mdpi.com/article/10.3390/microorganisms14092019/s1, Table S1: Complete in vitro probiotic-related functional properties of the 37 candidate strains.

## Abbreviations

The following abbreviations are used in this manuscript:

LAB	Lactic acid bacteria
MFS	Mixed fecal suspension
PCA	Principal component analysis
SCFA	Short-chain fatty acid
KMO	Kaiser–Meyer–Olkin
MRM	Multiple reaction monitoring
PCoA	Principal coordinates analysis
