# Supplementary material for: Sequential Screening of Lactic Acid Bacteria from Fermented Sour Bamboo Shoots and Their Effects on Patient-Derived Fecal Microbiota In Vitro
Source: Microorganisms. 2026 Sep 11;14(9):2019. doi: 10.3390/microorganisms14092019 (PMC13609094; doi:10.3390/microorganisms14092019)
Supplement: Supplementary file 1 [file microorganisms-14-02019-s001.zip › microorganisms-4530714-supplementary.pdf]

Sequential Screening of Lactic Acid Bacteria from Fermented Sour Bamboo Shoots and Their Effects on Patient-Derived Fecal Microbiota In Vitro

Junjie Li [1], Lianxian Ma [1], Rui Shi [1], Yuanrui Yu [1], Wenyan Zhang [1], Peng Zhu [1], Kaijie Kang [1,*], and Liwen Jiang [2,*]

1 School of Chemistry and Chemical Engineering, Zhaotong University, Zhaotong 657000, China; lijunjie900501@163.com (J.L.)

2 College of Food Science and Technology, Hunan Agricultural University, Changsha 410128, Hunan Province, China

* Correspondence: kangkaijie02@126.com (K.K.); hnndjlw@163.com (L.J.)

Supplementary Tables

Table S1. Complete in vitro probiotic-related functional properties of the 37 candidate strains

Strain	Ec-AA（cm）	Sd-AA（cm）	Vp-AA（cm）	GJ-SR（%）	SIF-SR（%）	Auto-C（%）	Co-C（%）	CSH（%）
MRS-1	0.63±0.05	1.23±0.10	0.24±0.04	72.54±1.35	16.41±1.06	42.32±3.32	13.32±0.39	75.55±3.45
MRS-6	0.83±0.04	1.53±0.13	0.55±0.08	79.33±1.06	51.28±0.38	45.12±1.87	17.65±0.80	78.77±5.46
MRS-12	0.75±0.26	1.55±0.09	0.48±0.05	78.43±1.21	48.28±1.72	43.33±2.39	23.32±1.11	85.33±2.54
MRS-13	0.45±0.15	0.83±0.11	0.24±0.04	70.26±0.46	31.80±0.47	22.30±2.55	14.34±0.46	89.06±0.97
MRS-14	0.67±0.08	0.88±0.23	0.16±0.06	71.35±1.34	26.49±1.22	40.35±1.23	21.36±0.85	45.44±3.21
MRS-16	0.46±0.15	1.47±0.14	0.34±0.04	72.40±1.21	13.45±0.86	42.32±3.37	17.43±1.13	44.30±2.467
MRS-18	0.33±0.05	0.69±0.07	0.38±0.07	70.87±0.87	14.43±0.16	37.18±2.18	18.67±0.85	67.98±3.60
MRS-19	0.44±0.07	1.26±0.03	0.37±0.06	72.75±1.54	16.44±0.20	42.64±1.06	17.85±0.26	54.34±5.77
MRS-22	0.58±0.15	0.62±0.12	0.42±0.08	72.87±0.56	17.64±0.09	43.22±1.42	15.85±0.93	79.65±4.58
MRS-27	0.70±0.07	1.33±0.04	0.24±0.06	77.67±1.16	50.32±1.65	50.34±0.45	24.76±1.68	83.62±4.67
MRS-34	0.73±0.06	1.33±0.06	0.62±0.02	77.42±1.22	50.30±1.48	46.47±1.06	24.02±1.03	78.35±4.29
MRS-36	0.68±0.07	1.60±0.20	0.40±0.07	77.75±0.08	40.41±1.40	39.22±3.65	28.76±1.56	67.85±3.61
MRS-46	0.61±0.04	1.53±0.15	0.64±0.08	76.08±1.20	46.21±0.66	54.12±1.40	26.76±1.12	69.46±1.40
MRS-49	0.43±0.03	0.98±0.03	0.37±0.08	75.77±1.16	15.25±1.38	46.36±2.08	19.65±1.60	89.65±7.56
MRS-51	0.43±0.04	0.73±0.09	0.54±0.07	71.96±0.84	10.40±1.85	40.30±3.87	26.68±1.28	76.65±3.38
M17-2	0.43±0.16	0.63±0.05	0.62±0.03	69.54±1.27	26.22±1.60	22.65±1.53	15.76±1.35	79.54±4.35
M17-3	0.56±0.06	1.45±0.05	0.55±0.09	70.32±1.20	14.35±1.46	34.52±3.79	21.43±1.06	50.69±3.07
M17-4	0.41±0.06	0.77±0.11	0.20±0.04	69.36±1.21	18.01±1.71	25.43±2.20	19.39±0.65	46.42±3.74
M17-5	0.67±0.05	0.68±0.16	0.67±0.08	76.39±1.22	8.75±1.26	27.34±2.52	17.35±0.34	64.17±5.20
M17-6	0.33±0.07	0.83±0.10	0.50±0.05	78.48±0.86	26.55±0.07	37.32±2.51	8.39±1.79	68.64±3.55
M17-7	0.46±0.06	0.93±0.06	0.28±0.09	71.76±1.56	30.48±2.06	41.36±1.23	22.76±0.80	69.15±5.65
M17-8	0.43±0.09	0.79±0.10	0.40±0.06	68.42±1.45	31.24±1.50	40.39±1.50	20.35±1.11	73.42±3.84

M17-10	0.49±0.06	0.85±0.17	0.34±0.09	71.58±2.04	11.42±0.47	41.36±2.05	24.76±0.90	66.77±3.54
M17-18	0.63±0.04	0.73±0.05	0.36±0.05	73.36±1.51	37.48±1.80	32.98±3.51	18.75±1.44	63.32±4.71
M17-22	0.83±0.03	1.13±0.20	0.53±0.08	79.21±1.36	42.91±1.90	49.13±3.40	16.35±1.14	64.27±3.10
M17-24	0.73±0.15	1.58±0.05	0.67±0.05	76.40±1.22	43.42±1.20	48.34±2.50	16.80±0.70	79.06±0.73
M17-29	0.63±0.06	1.23±0.14	0.39±0.08	66.43±1.36	19.87±2.01	45.75±2.62	8.80±0.97	68.94±3.03
M17-30	0.53±0.06	0.46±0.08	0.37±0.06	67.83±1.30	35.46±1.54	40.32±3.76	14.70±1.71	69.18±5.34
M17-36	0.48±0.07	0.73±0.07	0.30±0.05	74.42±0.26	13.44±0.97	37.38±2.65	15.46±1.59	40.65±3.16
M17-38	0.40±0.08	0.88±0.03	0.46±0.06	62.17±1.45	30.84±0.38	39.30±2.03	18.75±1.40	58.39±5.62
MC-1	0.50±0.10	0.76±0.15	0.55±0.04	63.99±0.86	35.74±1.10	41.33±1.05	17.76±1.42	64.26±4.53
MC-4	0.40±0.14	0.63±0.08	0.20±0.06	66.83±1.18	38.90±1.41	42.11±1.36	20.02±1.90	80.01±8.49
MC-5	0.36±0.08	0.98±0.15	0.30±0.04	74.28±0.88	35.47±1.90	47.31±1.60	21.20±1.30	48.44±6.42
MC-10	0.62±0.13	0.83±0.10	0.44±0.07	79.26±0.77	24.01±2.64	37.12±2.08	17.89±1.40	80.61±6.84
MC-19	0.63±0.05	0.60±0.10	0.28±0.09	66.49±1.38	21.11±1.20	44.38±1.43	20.49±0.75	70.94±5.66
MC-24	0.50±0.07	0.73±0.13	0.69±0.03	76.40±0.48	19.91±1.75	34.32±1.73	25.64±1.35	69.43±2.90
MC-38	0.70±0.16	1.52±0.11	0.53±0.02	75.93±0.96	40.05±2.01	41.33±2.61	24.28±1.38	86.75±6.28
